# Combination of Ruthenium Complex and Doxorubicin Synergistically Inhibits Cancer Cell Growth by Down-Regulating PI3K/AKT Signaling Pathway

**DOI:** 10.3389/fonc.2020.00141

**Published:** 2020-02-18

**Authors:** Ke Lin, Yi Rong, Dan Chen, Zizhuo Zhao, Huaben Bo, Aimin Qiao, Xiaojuan Hao, Jinquan Wang

**Affiliations:** ^1^Guangdong Province Key Laboratory for Biotechnology Drug Candidates, School of Bioscience and Biopharmaceutics, Guangdong Pharmaceutical University, Guangzhou, China; ^2^Department of Ultrasound, Sun Yat-sen Memorial Hospital, Sun Yat-sen University, Guangzhou, China; ^3^Manufacturing, Commonwealth Scientific and Industrial Research Organisation (CSIRO), Clayton, VIC, Australia

**Keywords:** ruthenium complex, doxorubicin, combination therapy, synergistic effect, PI3K/AKT signaling pathway

## Abstract

Combinational use of drugs has been a common strategy in cancer treatment because of synergistic advantages in reducing dose and toxicity, minimizing or delaying drug resistance. To improve the efficacy of chemotherapy, various potential combinations have been investigated. Ruthenium complex is considered a potential alternative of the platinum-based drugs due to its significant efficacy and safety. Previously, we reported that ruthenium(II) complex (Δ-Ru1) has great anticancer potential and minor toxicity toward normal tissues. However, the therapeutic efficacy and mechanism of action of ruthenium(II) complex combined with other anticancer drugs is still unknown. Here, we investigated the combinational effect of Δ-Ru1 and doxorubicin in different cancer cells. The data assessed by Chou-Talalay method showed significant synergism in MCF-7 cells. Furthermore, the results in antiproliferation efficacy indicated that the combination showed strong cytotoxicity and increasing apoptosis of MCF-7 cells in 2D and 3D multicellular tumor spheroids (MCTSs). Significant inhibition of MCF-7 cells accompanied with increased ROS generation was observed. Furthermore, the expression of PI3K/AKT was significantly down-regulated, while the expression of PTEN was strongly up-regulated in cells treated with combination of Δ-Ru1 and doxorubicin. The expression of NF-κB and XIAP decreased while the expression of P53 increased and associated with apoptosis. These findings suggest that the combination of ruthenium complex and doxorubicin has a significant synergistic effect by down-regulating the PI3K/AKT signaling pathway in MCF-7 cells. This study may trigger more research in ruthenium complex and combination therapy that will be able to provide opportunities for developing better therapeutics for cancer treatment.

## Introduction

Combinational use of drugs is a common strategy in practice. The benefits of combinational use of drugs include synergistic effect, low toxicity, dosage reduction, and reduced drug resistance (1). For these reasons, combinational use of drugs is widely used in various diseases such as cancer, acquired immune deficiency syndrome, and other diseases (2). Chemotherapy plays a major role in cancer treatment. Since the development of cisplatin, platinum-based drugs have showed potent efficacy in anticancer treatment and many metallodrugs have been developed (3). However, the platinum-based complexes have severe side effects, including myelotoxicity, neurotoxicity, nephrotoxicity, and hair loss (4). Besides, drug resistance also limits the application of these drugs in clinics (5). To overcome these problems, the search for the alternatives of platinum complex and feasible drugs combination strategies has been performed.

The ruthenium complexes, as a new class of non-platinum metal complexes, are promising alternatives to platinum-based chemotherapeutic drugs because of their unique biochemical properties (6). It was reported that ruthenium complexes showed excellent anticancer efficacy in various types of cancers, and many of them have a better effect than cisplatin (7). Four of the ruthenium complexes, NAMI-A, KP1019, KP1339, and TLD1443, have been used in different stages of clinical trials and showed a good anticancer potential (8–11). Ruthenium-based complexes have more properties compared with platinum-based drugs, including variable oxidation states, better tumor selectivity, and similar ligand exchange kinetics (12). More importantly, ruthenium-based complexes showed mild toxicity toward normal tissues, making them promising chemotherapeutic agents (13). The application of ruthenium complex is vast because of its special structure. The anticancer potential of ruthenium complexes is associated with targets and signaling pathways and depends on the properties of the complex, the ligands, and the sites of tumors (14). For example, ruthenium complexes induced cell death might involve the mitochondria-mediated signaling pathway, the death receptor-mediated signaling pathway, and the endoplasmic reticulum signaling pathways (15–18). Accordingly, the various targets of ruthenium complexes for cell death are mitochondria, endoplasmic reticulum, and multiple molecules (19–23). In addition, some ruthenium complexes can act as radiosensitizers, theranostic agents, photothermal therapy (PTT) agents, and photodynamic therapy (PDT) agents (24, 25). The multifunctional ruthenium complexes can be combined with other therapies, such as radiotherapy, nanotechnology, and targeted therapy (26, 27). The bioavailability of ruthenium complexes can be effectively improved by these combination strategies for better anticancer effect.

In preclinical studies, the ruthenium complexes combined with different anticancer agents have been investigated. The efficacy of anticancer agents combined with the ruthenium complexes have showed significant outcomes. In a previous study, the KP1339/sorafenib combination showed a great activity *in vitro* and *in vivo* (28). It was reported that the NAMI-A/cisplatin combination showed additive effect compared with each drug taken alone (29). In another preclinical study, it was found that NAMI-A and doxorubicin have a synergistic effect on lung metastasis in a mouse model. However, there was a high toxicity when these two drugs were taken at the maximum tolerated doses (30). Therefore, more studies are needed on the combined action of ruthenium complexes with other anticancer drugs.

In this study, we investigated the efficacy and potential mechanism of the combinational use of ruthenium(II) complex (Δ-Ru1) and doxorubicin (Dox). We found that the Δ-Ru1/Dox combination has a strong synergistic effect in inhibiting the growth of MCF-7 cells with great combination indexes (CI). According to the Chou-Talalay method, CI between 0 and 1 indicate synergism and the smaller CI means the stronger synergism (31). In addition, the Δ-Ru1/Dox combination inhibited the proliferation of multicellular tumor spheroids (MCTSs). Furthermore, we found that the Δ-Ru1/Dox combination enhanced cellular apoptosis and increased ROS generation. The Western blot analysis suggested that the synergistic effect of Δ-Ru1/Dox combination is regulated by the PI3K/AKT pathway, and is associated with the expression of PTEN, NF-κB, XIAP, and P53.

## Materials and Methods

### Materials

Ruthenium(II) complex (Δ-[Ru(bpy)_2_(HPIP)](ClO_4_)_2_) (Δ-Ru1) was prepared as our previous study (32). Doxorubicin (CAS: 25316-40-9) was purchased from Energy Chemical (Shanghai, China). Δ-Ru1 and doxorubicin dissolved in dimethyl sulfoxide (DMSO) to 10 mM for stock solution. Both drugs were stored at −20°C and diluted with PBS before use. Fetal bovine serum (FBS) and RPMI-1640/DMEM medium were purchased from Gibco (BRL, Grand Island, NY). Hoechst 33342, 3-(4,5-dimethylthiazol-2-yl)−2,5-diphenyl tetrazolium bromide (MTT), DMSO were obtained from Sigma-Aldrich (St. Louis, MO, USA). The Annexin V-FITC apoptosis assay kit, ROS detection kit, and Calcein AM detection kit were obtained from Life Technologies (BRL, Grand Island, NY).

### Cell Culture

Cancer cell lines SW116, H1299, B16F10, HepG-2, MCF-7, and MDA-MB-231 were obtained from Sun Yat-Sen University (Guangzhou, China). B16F10 cells were cultured in 1,640 medium, all other cells were cultured in DMEM (with 10% FBS). Cells were cultured in a humidified atmosphere with 5% CO_2_ at 37°C.

### Cytotoxicity Assay and Combination Index

The MTT assay was performed to detect the cell viability as described previously (33). Cells at the logarithmic growth phase were seeded in a 96-well plate and incubated for 24 h. Δ-Ru1 (5, 10, 20, 40, 80, 160, 320 μM) and Dox (0.5, 1, 2, 4, 8, 16, 32 μM) were added and incubated for 24 h. Replaced medium (with 10% FBS) and 20 μL of MTT solution (5 mg/ml in PBS) were added to each well and incubated for 4 h. After that, the medium was removed and 200 μL DOSO was added to dissolve blue-violet crystals by shaking gently for 10 min. The optical density (OD) of each well was then measured on a multifunction full wavelength scanner (Biorad, USA) at a wavelength of 570 nm. Cell Viability (%) = (OD_Drug_ – OD_Blank_)/(OD_Control_ – OD_Blank_) × 100%. IC_50_ values were calculated with SPSS 22. The combination effect was analyzed with Compusyn software (Biosoft, Inc., MO, USA). According to the quantitative determination, the combination index (CI) was calculated. The drug combination is considered as synergism if CI <1, antagonism if CI > 1, and additive effect if CI = 1 (31).

### Real-Time Cell Growth and Proliferation Assay

Experiments were carried out as described previously, using an xCELLigence RTCA DE System (Roche Diagnostics GmbH, Germany) (34). Briefly, 100 μL of cell culture medium was added to E-plate 16 (Roche Diagnostics GmbH, Germany), connected to the system, and the background impedance was measured. Meanwhile, the MCF-7 cells were adjusted to 1 × 10^4^ cells/well. Approximately 24 h after seeding, the cells were exposed to 100 μL of medium, with the ultimate concentration of Δ-Ru1 (20 μM) and Dox (2 μM). The cells were monitored for 24 h. The cellular proliferation index was recorded every 15 min using an RTCA analyzer. The cell index values were normalized to the value before treatment to eliminate the variation between the wells. Data were collected by the RTCA software supplied with the instrument.

### Apoptosis Detection

The early-phase apoptosis of MCF-7 cells was detected by the Annexin V staining and flow cytometry. MCF-7 cells were adjusted to a density of 1 × 10^5^ cells/well, seeded in six-well plates, and incubated overnight. Drugs were added as described above and incubated for 24 h. The cells were trypsinized, washed twice with PBS, and resuspended in 100 μL of binding buffer containing 5 μL of Annexin V stock solution (Invitrogen, Paisley, UK). The cells were incubated for 30 min at room temperature in the dark. The samples were quantified by flow cytometry (FACSCanto II, BD Biosciences, San Jose, CA, USA). The data were acquired and analyzed using FlowJo V10.

The late-phase apoptosis was detected with Hoechst staining, which is a common method to characterize apoptosis (35). MCF-7 cells were prepared as described earlier, and treated with the drugs for 24 h, washed twice with PBS before incubating with Hoechst 33342 (10 μg/ml) for 10 min. The Hoechst-stained cells were washed twice with PBS before the samples were observed under an inverted fluorescence microscope (Zeiss, Model Axio Observer D1, Germany).

### Live/Death Viability Assay in 2D MCF-7 Cell and 3D Multicellular Tumor Spheroids

Calcein AM staining assay was conducted to detect the live/death viability of MCF-7 cells after drug treatment. Cells were washed twice with PBS before incubating with Calcein AM for 30 min at 37°C in a 5% CO_2_ incubator. Then cells were washed twice with PBS before the samples were observed under an inverted fluorescence microscope.

The multicellular tumor spheroids (MCTSs) imitating solid tumors were usually used to verify the potency of the drugs. Live cells were distinguished by the presence of intracellular esterase, which can convert the non-fluorescent cell-permeant Calein AM into the green fluorescent calcine (36). The multicellular tumor spheroids (MCTSs) were cultured as previous study (37). Briefly, 1 × 10^4^ cells suspended in 100 μL of medium were added into a 96-well plate (Corning Spheroid Microplate). The cells were incubated for 72 h until the spheroids formed. After the formation of MCTSs, the drugs were given as described above for 24 h. The medium was removed and replaced by a new medium containing Calcein AM. MCTSs were incubated at 37°C in a 5% CO_2_ incubator for 30 min before washing twice with PBS, and then the samples were imaged under an inverted fluorescence microscope (λ ex = 488 nm, λ em = 520 ± 20 nm). In another assay, the MCTSs were cultured as described above, and incubated with drugs for 9 days to investigate the size change of MCTSs. The new medium was replaced every 3 days and each MCTSs in a 96-well plate was measured with a phase-contrast microscope to monitor the diameter of the spheroids. The relative diameter of MCTSs was calculated by V_t_/V_0_, where V_t_ is the MCTSs diameter on the day of the drug treatment, V_0_ is the control MCTSs diameter on the day of the initial treatment.

### Reactive Oxygen Species (ROS) Measurement

MCF-7 cells and multicellular tumor spheroids (MCTSs) were prepared as above. After cells were treated with the drugs for 24 h, MCF-7 cells and MCTSs were incubated in 10 μM DCFH-DA containing medium at 37°C for 30 min. DCFH-DA is a non-fluorescent complex that becomes the highly green fluorescent 2′,7′-dichlorofluorescein on oxidation by intracellular ROS. The samples were then washed twice with PBS to remove DCFH-DA before the fluorescence was visualized by an inverted fluorescence microscope (Zeiss, Model Axio Observer D1, Germany).

### Western Blot Analysis

For whole-cell protein extracts, cells were treated with drugs for 24 h. Cells were washed twice with ice-cold PBS and lysed on ice with radio immune precipitation assay buffer (RIPA) containing protease and phosphatase inhibitor cocktails (MedChemExpress) to extract the total proteins. Equal amounts of proteins were separated with sodium dodecyl sulfate-polyacrylamide gels electrophoresis (6 or 10%). Next, the proteins were transferred to PVDF membranes (Millipore, Bedford, MA, USA), which were then blocked at room temperature for 1 h with 5% non-fat milk. Membranes were incubated overnight with non-conjugated primary antibodies against GAPDH (10494-1-AP) (Proteintech, MA, USA), PTEN (#9188), PI3K (#4249), P-AKT (Ser 473) (#4060), NF-κB (#8242), P-NF-κB (Ser 536) (#3033), XIAP (#2045), P53 (#2527) from Cell Signaling Technology (Danvers, MA, USA), and P-PI3K (110α) (11508) and AKT (21055) from Signalway Antibosy (College Park, Maryland, USA). Membranes were then incubated with the labeled secondary antibody horseradish peroxidase (HRP) conjugated goat anti-rabbit IgG antibodies (SA00001-2) from Proteintech (MA, USA). The enhanced chemiluminescence (ECL) detection system was used to detect the reactive protein bands, that were developed on film in a dark room.

### Statistical Analysis

Data were reported as the mean ± standard deviation. Each experiment was statistically analyzed using the *t*-test for grouped data. *P* < 0.05 was considered significant.

## Results

### The Inhibitory Effect of Δ-Ru1 and Doxorubicin in Cancer Cells

**Figure 1:**
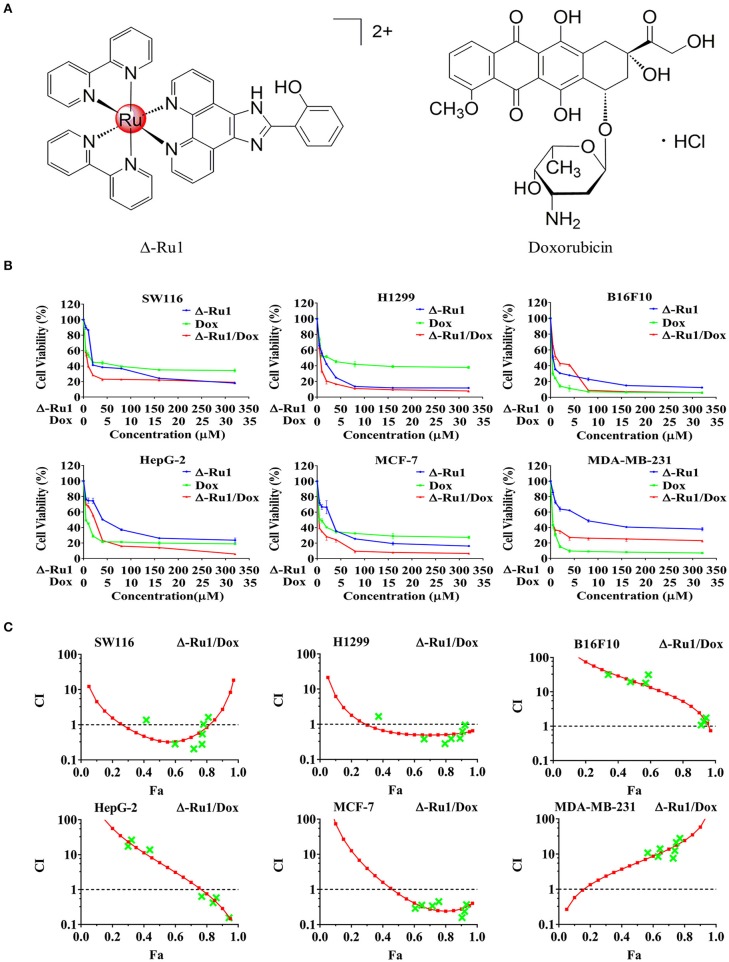
The cytotoxicity of the Δ-Ru1/Dox combination in different cancer cells and the combination index. **(A)** The chemical structures of Δ-Ru1 and Doxorubicin. **(B)** Cells viability under the treatment with Δ-Ru1/Dox and determined by MTT assay. Cancer cells (SW116, H1299, B16F10, HepG-2, MCF-7, and MDA-MB-231) were treated with or without Δ-Ru1 (5, 10, 20, 40, 80, 160, 320 μM), Dox (0.5, 1, 2, 4, 8, 16, 32 μM), and the combination of Δ-Ru1/Dox (10:1) for 24 h. **(C)** Combination index (CI)-fraction affected (Fa, corresponding to the fraction of cell viability) plot of the Δ-Ru1/Dox combination in different cancer cells assessed with Compusyn Software.

Ruthenium(II) complex (Δ-[Ru(bpy)_2_(HPIP)](ClO_4_)_2_) (Δ-Ru1) has been reported in our previous study, which shows a great anti-cancer efficacy (32). In this study, we investigated the tumor inhibitory effect of Δ-Ru1 in combination with doxorubicin (Dox) (Figure 1A). We detected enhanced cytotoxic effect of the Δ-Ru1/Dox combination in six selected cancer cell lines (SW116, H1299, B16F10, HepG-2, MCF-7, and MDA-MB-231) compared with Δ-Ru1alone and Dox alone. The results of cell viability were shown in Figure 1B and IC_50_ shown in Table 1. The results indicated that different cell lines showed different IC_50_ to Δ-Ru1 (from 3.9 ± 1.0 to 88.3 ± 13.4 μM). In contrast, cancer cells were more sensitive to Dox, which have smaller IC_50_ (from 0.7 ± 0.9 to 3.5 ± 1.4 μM). When these two drugs were combined, the cytotoxic potency increased. Notably, the cytotoxicity of the Δ-Ru1/Dox combination to H1299 and MCF-7 cells were stronger than Δ-Ru1 alone or Dox alone at all fractions affected (Fa). The cytotoxicity of Δ-Ru1/Dox combination to these cancer cells was in a dose-dependent manner and their anticancer efficacy is most prominent in MCF-7 cells.

**Table 1:** IC_50_ values of Δ-Ru1 and Dox in selected cancer cells.

**Cell line**	**IC_50_ (μM)**		
	**Δ-Ru1**	**Dox**	**Δ-Ru1/Dox**
SW116	40.2 ± 8.1	1.6 ± 0.4	4.4 ± 0.3
H1299	11.4 ± 1.3	3.5 ± 1.4	5.2 ± 0.6
B16F10	3.90 ± 1.0	0.7 ± 0.9	13.5 ± 0.9
HepG-2	45.5 ± 7.4	1.3 ± 0.2	18.5 ± 0.9
MCF-7	24.0 ± 4.9	2.1 ± 0.2	3.4 ± 0.6
MDA-MB-231	88.3 ± 13.4	1.0 ± 0.4	1.2 ± 0.2

*Data are expressed as Mean ± SD (n = 3)*.

To detect the efficacy of the Δ-Ru1/Dox combination, the combination index (CI) plots of these drugs were generated using the Compusyn software. Figure 1C and Table 2 showed the CI value of the Δ-Ru1/Dox combination in six different cancer cells. The combination of Δ-Ru1 and Dox showed antagonistic effect in B16F10 and MDA-MB-231 cells (CI > 1). In HepG-2 cells, the Δ-Ru1/Dox combination was antagonistic for Fa lower than 77% but synergistic above this value. In H1299 cells, the Δ-Ru1/Dox combination was synergistic for nearly all Fa. While in SW116, this combination was synergistic only at Fa <0.8. Notably, this combination was synergistic in MCF-7 cells and had CI <0.3 at nearly all Fa, which means strong synergism according to Chou-Talalay method (31).

**Table 2:** The synergistic effect of the Δ-Ru1/Dox combination in selected cell lines.

**Δ-Ru1**	**Dox**	**SW116**		**H1299**		**B16F10**	
		**Fa**	**CI**	**Fa**	**CI**	**Fa**	**CI**
5	0.5	0.42	0.36	0.37	1.67	0.34	31.56
10	1	0.60	0.28	0.66	0.38	0.48	19.48
20	2	0.72	0.21	0.79	0.28	0.57	17.99
40	4	0.77	0.28	0.83	0.39	0.59	31.35
80	8	0.77	0.55	0.89	0.40	0.91	1.08
160	16	0.78	1.03	0.91	0.63	0.93	1.37
320	32	0.81	1.62	0.92	0.95	0.94	1.75
**Δ-Ru1**	**Dox**	**HepG-2**		**MCF-7**		**MDA-MB-231**	
		**Fa**	**CI**	**Fa**	**CI**	**Fa**	**CI**
5	0.5	0.30	17.32	0.61	0.29	0.57	10.82
10	1	0.32	26.26	0.65	0.36	0.63	8.50
20	2	0.44	13.76	0.72	0.34	0.64	14.10
40	4	0.77	0.64	0.76	0.45	0.73	7.60
80	8	0.84	0.42	0.90	0.16	0.74	12.59
160	16	0.86	0.59	0.92	0.24	0.75	21.31
320	32	0.94	0.16	0.93	0.37	0.77	27.99

*The concentration of Δ-Ru1 is 5, 10, 20, 40, 80, 160, 320 μM. The concentration of doxotubicin (Dox) is 0.5, 1, 2, 4, 8, 16, 32 μM. Fa, fraction affected, corresponding to the fraction of cell viability (%); CI, combination index. CI <1 indicate synergism, CI > 1 indicate antagonism, and CI = 1 indicate additive effect*.

**Figure 2:**
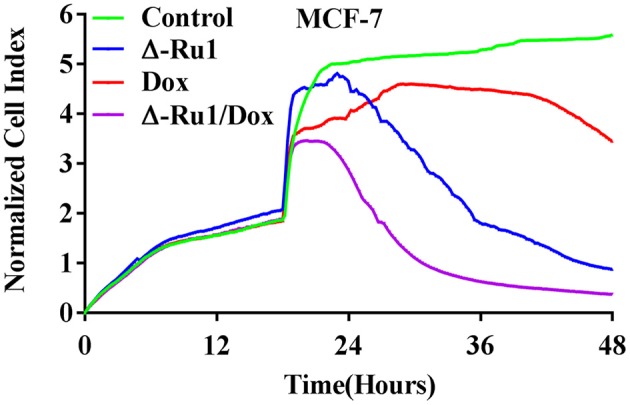
A real-time cell analysis for the Δ-Ru1/Dox combination in MCF-7 cells monitored by the xCELLigence system. MCF-7 cells were incubated with Δ-Ru1 (20 μM) alone, Dox (2 μM) alone or the Δ-Ru1/Dox combination for 24 h. The kinetics of cytotoxicity responses were collected as cell index.

Furthermore, we investigated the synergistic effect of the Δ-Ru1/Dox combination with a real-time cell analysis. The kinetic profiles detected by the xCELLigence system indicated that the dynamics of cytotoxicity were different among Δ-Ru1, Dox, and the Δ-Ru1/Dox combination (Figure 2). The Δ-Ru1 appears to take a longer time to manifest its effect, while Dox has a mild effect on the cells. Notably, the Δ-Ru1/Dox combination rapidly abolished cellular proliferation, suggesting that cells undergo irreversible inhibition. MCF-7 cells exhibited the highest sensitivity to drug combination, which was consistent with the results of MTT assay and showed a predictable synergistic effect of the Δ-Ru1/Dox combination. These results suggested that the Δ-Ru1/Dox combination has a great synergistic effect on MCF-7 cells. Thus, we chose MCF-7 cells for further experiments.

### Antiproliferation Effect of the Δ-Ru1/Dox Combination in Cancer Cells and Multicellular Tumor Spheroids

**Figure 3:**
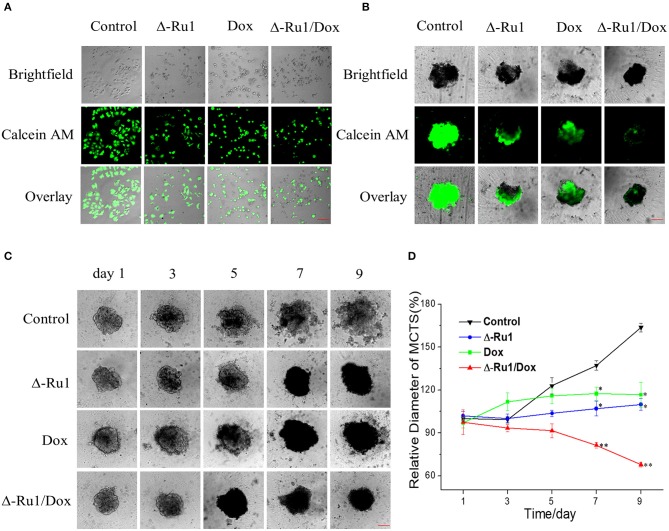
The antiproliferative effect of the Δ-Ru1/Dox combination in MCF-7 cells and multicellular tumor spheroids (MCTSs). Cells were treated with Δ-Ru1 (20 μM) alone, Dox (2 μM) alone or in combination for 24 h. **(A)** Viability of MCF-7 cells observed by fluorescence microscopy after incubating with Calcein AM (λ ex = 488 nm, λ em = 520 ± 20 nm). *Scale bar 200* μ*m*. **(B)** MCF-7 MCTSs imaged with the dye Calcein AM after drug treatment for 24 h. MCF-7 cells in a total of 1 × 10^4^ cells cultured for 72 h. *Scale bar 200* μ*m*. **(C)** MCTSs incubated with the drug by increasing days. The MCTSs incubated with Δ-Ru1 (20 μM) alone, Dox (2 μM) alone, or the Δ-Ru1/Dox combination observed under fluorescence microscope. *Scale bar 200* μ*m*. **(D)** Relative diameter change of MCTSs. The MCTSs diameter on the day of the initial treatment was set as 100%. *p < 0.05, **p < 0.01.

To find out if the cancer cell inhibitory effect of the Δ-Ru1/Dox combination is associated with antiproliferation, we used Calcein AM staining in MCF-7 cells and multicellular tumor spheroids (MCTSs). Calcein AM stained the viable cells with green fluorescence in MCF-7 cells (Figure 3A). Notably, the results indicated that the Δ-Ru1/Dox combination had a stronger antiproliferation effect in MCF-7 cells compared with Δ-Ru1 alone and Dox alone. MCTSs are multicellular aggregates and have been gradually accepted as a valid 3D cancer model in reproducing the complexity and pathophysiology of *in vivo* solid tumors (36). As expected, the cells of the untreated MCTS were all alive, as indicated by the strong green fluorescence. However, the weakening green fluorescence was observed in MCTSs treated with Δ-Ru1 alone and Dox alone. Furthermore, the green fluorescence in MCTSs treated with Δ-Ru1/Dox combination can hardly be detected (Figure 3B).

In addition, we observed the similar synergistic effect on the kinetics of 3D tumor growth. After treatment with Δ-Ru1 alone and Dox alone, the size of MCTSs still increased, although the growth rate is much lower than that of the control group. However, the diameter of MCTSs treated with the Δ-Ru1/Dox combination became 68% of the initial diameter of the control group after 9 days, indicating that the Δ-Ru1/Dox combination had the strongest antiproliferative activity against MCF-7 MCTSs (Figures 3C,D). These data indicated that the Δ-Ru1/Dox combination exhibited a synergistic therapeutic effect in MCF-7 cells and MCTSs.

### Δ-Ru1 and Doxorubicin Combination Enhanced Cell Apoptosis

**Figure 4:**
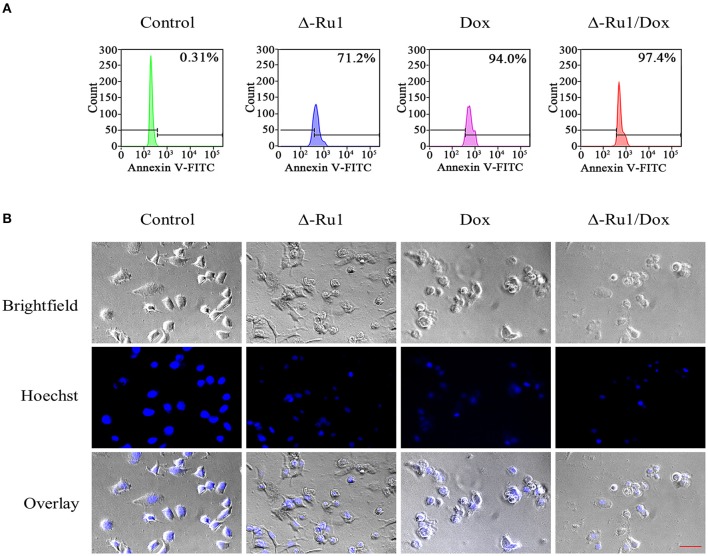
The Δ-Ru1/Dox combination increased apoptosis in MCF-7 cells. Cells were treated with Δ-Ru1 (20 μM) alone, Dox (2 μM) alone or in combination for 24 h. **(A)** Early-phase apoptosis in MCF-7 cells determined by flow cytometry after staining with Annexin V-FITC. **(B)** Late-phase apoptosis examined by analyzing nuclear fragmentation with Hoechst staining and observed by fluorescence microscopy. *Scale bar 200* μ*m*.

We further investigated if the inhibitory effect of Δ-Ru1/Dox combination was associated with cell apoptosis. We performed Annexin V-FITC staining and flow cytometry analysis. The results showed that the drug treatment resulted in significant apoptosis compared with the control group (Figure 4A). In addition, the early-phase apoptosis induced by the Δ-Ru1/Dox combination was more significant than Δ-Ru1 alone and Dox alone. Hoechst staining was used to detect late-phase apoptosis. As expected, after exposure to drugs for 24 h, the cell nuclei shrank or disappeared, and this phenomenon was more severe in the Δ-Ru1/Dox combination compared with Δ-Ru1 alone and Dox alone, whereas the untreated cells remained the normal shape (Figure 4B). These results indicated that the Δ-Ru1/Dox combination effectively enhanced the cytotoxic effects on MCF-7 cells by inducing cell apoptosis.

### Reactive Oxygen Species (ROS) Release

**Figure 5:**
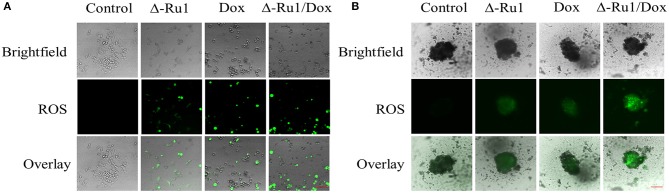
ROS generation in MCF-7 cells and multicellular tumor spheroids (MCTSs). Cells were treated with Δ-Ru1 (20 μM) alone, Dox (2 μM) alone or in combination for 24 h. **(A)** ROS in 2D MCF-7 cells was determined by staining with DCFH-DA and observed by fluorescence microscopy. *Scale bar 200* μ*m*. **(B)** ROS in MCTSs. *Scale bar 200* μ*m*.

Ruthenium complex usually triggers reactive oxygen species (ROS) accumulation in cancer cells (38). To investigate the status of ROS generation, we measured it in MCF-7 cells and MCTSs. The data showed an increased ROS accumulation in MCF-7 cells after treatment compared with the control group, which had none of the green fluorescence being observed (Figure 5A). Cells in the Δ-Ru1/Dox combination generated the strongest green fluorescence, compared with Δ-Ru1 alone and Dox alone. Furthermore, ROS generation in MCTSs was showed in Figure 5B. As expected, the data in MCTSs were consistent with that in MCF-7 cells and indicated that the Δ-Ru1/Dox combination generated the highest ROS accumulation, which is associated with apoptosis.

### The Change of Molecules Associated With Apoptotic Pathways in Cells Treated With Δ-Ru1/Dox Combination

**Figure 6:**
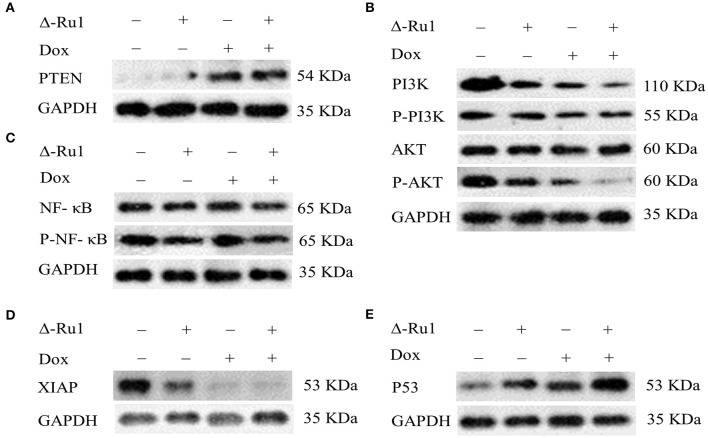
Western blotting for PTEN, PI3K (P-PI3K 110α), AKT (P-AKT, Ser 473), NF-κB (P-NF-κB, Ser 536), XIAP, and P53. Cells were treated with Δ-Ru1 (20 μM) alone, Dox (2 μM) alone or in combination for 24 h, and immunoblotted with the indicated antibodies. P- represent phosphorylation. **(A)** The expression of PTEN. **(B)** The expression of PI3K/P-PI3K and AKT/P-AKT. **(C)** The expression of NF-κB and P-NF-κB. **(D)** The expression of XIAP. **(E)** The expression of P53.

Finally, we investigated the molecules that are related to cell proliferation and survival pathways upon the treatment with the Δ-Ru1/Dox combination in MCF-7 cells. We focused our study on the PI3K/AKT signaling pathway, which was associated with proliferation and survival of MCF-7 cells (39). We also detected PTEN and NF-κB, which are the key members of the PI3K/AKT signaling pathway (40, 41). In addition, the signals regulated by the Δ-Ru1/Dox combination were detected, including XIAP and P53, which are associated with the intrinsic apoptotic pathway (42, 43). The Western blot results showed that the Δ-Ru1/Dox combination up-regulated PTEN expression compared with Δ-Ru1 alone and Dox alone, whereas PTEN was absent in the control group (Figure 6A). The expression of PI3K and AKT decreased in the treatment of Δ-Ru1 alone, Dox alone or with the Δ-Ru1/Dox combination. The Δ-Ru1/Dox combination showed the greatest down-regulation of phosphorylated PI3K/AKT in the MCF-7 cells (Figure 6B). Nuclear factor κB (NF-κB) was known as an important regulator in cell apoptosis, which is associated with the function of AKT. As shown in Figure 6C, NF-κB expressed in all treatment groups. Besides, the expression of phosphorylated NF-κB showed a slight inhibition in Δ-Ru1, Dox, and the Δ-Ru1/Dox combination compared with the control group, which has the highest level of expression. The expression of XIAP under the treatment showed that cells treated with Δ-Ru1 alone, Dox alone, and the Δ-Ru1/Dox combination greatly inhibited XIAP expression (Figure 6D). MCF-7 cells incubated with Dox or the Δ-Ru1/Dox combination induced similar XIAP expression at an extremely low level compared with the control group, which had the highest expression. Furthermore, the expression of P53 was greatly up-regulated in cells treated with Δ-Ru1, Dox, and the Δ-Ru1/Dox combination, compared with the control group. Notably, a significant up-regulation of P53 was seen in the Δ-Ru1/Dox combination group, and its expression was higher than that in cells treated with Δ-Ru1 alone and Dox alone (Figure 6E).

## Discussion

Tumor inhibition and elimination is the goal of clinical treatment, in which chemotherapy plays a significant role. There are disadvantages of single chemotherapeutic agent, and combinational use of drugs are widely used in the clinics. Effective drug combination is critical. In previous studies, platinum-based drugs in combination with other chemotherapeutic agents showed favorable anticancer activity, which includes synergistic effect, dose reduction, and reducing the risk of resistance (44, 45). However, the application of these combinations was limited by the toxicity of platinum-based drugs. Fortunately, the ruthenium complex, as a promising alternative to platinum-based drugs, has attracted great interest in the field. Numerous studies have demonstrated that the ruthenium complex has a great anticancer potential, and favorable outcomes of combination therapy (46–48). Furthermore, in preclinical studies, ruthenium complexes NAMI-A and KP1339 combined with other chemotherapeutic drugs have shown synergistic effects (28–30). However, the efficacy and mechanism of the combinational use of ruthenium complex and other anticancer agents are largely unknown and further studies are needed.

In the present studies, we investigated the effect of combinational use of a ruthenium(II) complex (Δ-Ru1) and doxorubicin (Dox) in cancer cells. Our data showed that the Δ-Ru1/Dox combination had a strong synergistic effect against the growth of MCF-7 cells. The synergism and antagonism were quantitatively determined by the combination index theorem and the median-effect equation (MEE) of the mass-action law, which is a simple, efficient, and low-cost way to assess the combination effect between drugs and widely used around the world (49). To further confirm the synergistic effect of Δ-Ru1/Dox combination, we conducted a real-time cell analysis, and the results suggested that the Δ-Ru1/Dox combination has the strongest antiproliferation effect on MCF-7 cells, compared with Δ-Ru1 alone and Dox alone. The *in vitro* screening of anticancer drugs is usually performed in a two-dimensional (2D) level. However, it is limited in simulating the biological properties of *in vivo* solid tumors. Multicellular tumor spheroids (MCTSs) are aggregates of heterogeneous cells, which have been widely accepted and used as a valid 3D cancer model between 2D monolayer cells and solid tumors (50). MCTSs reproduce the biological properties of solid tumors and reflect cellular heterogeneity, such as cell-cell matrix interactions, nutrient and oxygen gradients, hypoxic/necrotic regions, and gene expression (51). Therefore, to examine the anticancer activity of Δ-Ru1 and Dox, in a more biologically relevant and complicated conditions, the antiproliferation assay was examined in a 3D tumor model by culturing MCF-7 MCTSs, compared with 2D MCF-7 cells model. The results showed that the antiproliferation effect in 3D MCTSs is consistent with the 2D level and the Δ-Ru1/Dox combination showed the strongest efficacy both in 2D and 3D levels. Notably, the MCTSs shrank to a smaller size after treatment with the Δ-Ru1/Dox combination for 9 days, which is considered as a favorable outcome in cancer therapy. These results further demonstrated the synergistic effect of Δ-Ru1/Dox combination in inhibiting MCF-7 cells.

Apoptosis is a major reason in cell death. Two classical pathways were involved in the induction of cellular apoptosis: the mitochondrial intrinsic pathway and the extrinsic death receptor pathway (52). To investigate if the anticancer effect of Δ-Ru1/Dox combination was induced by apoptosis, we examined the apoptosis in MCF-7 cells. The results suggested that the Δ-Ru1/Dox combination significantly enhanced apoptosis in MCF-7 cells. In addition, reactive oxygen species (ROS) are byproducts of the reduction of molecular oxygen formed in biological systems, which play a significant role in apoptosis. The endogenous sources of ROS include the mitochondrial electron transport chain and the cytochrome P450 CYP-dependent electron transport system (53). The oxygenation of the organic substrates and the reduction of molecular oxygen are catalyzed by the CYP enzymes. ROS is generated when the transfer of oxygen is out of control and uncoupling occurs (54). Intracellular biochemical antioxidants are usually maintained in balance with intracellular ROS, preventing cellular damage when a critical disruption occurred, and then oxidative stress eventually leads to apoptosis (55). Ruthenium complex induced ROS accumulation has been reported in previous studies, but whether the Δ-Ru1/Dox combination increases ROS generation was unknown. As expected, ROS accumulation in cells treated with the combination of Δ-Ru1/Dox combination was significantly increased in our results, which suggested the apoptosis enhancement by the treatment with the ΔRu1/Dox combination.

The phosphatidylinositol 3-kinase (PI3K)/AKT pathway, which regulates various cellular processes, including survival, proliferation, growth, metabolism, angiogenesis, and metastasis, had been well-demonstrated in previous studies (56). The hyperactivated PI3K/AKT signaling pathway has been found to be associated with the pathogenesis of breast cancer, which was implicated in conferring resistance to chemotherapy (57). In contrast, PTEN negatively regulates the PI3K/AKT signaling pathway, which is usually absent in breast cancer (58). To investigate whether the apoptosis enhancement is associated with this signaling pathway, we examined the expression of these proteins. Interestingly, we observed that the expression of PTEN was significantly up-regulated in the Δ-Ru1/Dox combination, while PI3K and AKT was down-regulated. NF-κB as a downstream target of AKT, was found to activate NF-κB by phosphorylating IκB kinase (IKK) to release transcription factor NF-κB from its sequestering IκB protein (59). The hyper-activation of NF-κB was usually detected in breast cancer, which has been reported to cause apoptosis inhibition (60). In the present study, the expression of NF-κB was down-regulated in cells treated with the Δ-Ru1/Dox combination, which may contribute to apoptosis.

The inhibitor of apoptosis proteins (IAP) is a family of endogenous antiapoptotic proteins. X-chromosome-linked IAP (XIAP), one of the eight human IAP proteins, has been found to exert the most pronounced antiapoptotic function, which is associated with its ability to block caspase−3, −7, and −9 (61). Moreover, some studies have demonstrated that XIAP has also been implicated in the regulation of NF-κB (62). In a previous study, it was found that Δ-Ru1 induces apoptosis through the mitochondrial-mediated pathway, and regulates the expression of proapoptotic proteins, including the down-regulation of Bcl-2/Bcl-xl, and the up-regulation of cytochrome c (Cyt C), caspase 9 and caspase 3 (32). Furthermore, the major mechanism of doxorubicin is the DNA damage response (DDR), which showed a strong cytotoxicity in cancer cells (63). P53 has been demonstrated to play a significant role in cancer treatment. The P53-mediated apoptosis had been implicated in an ability to suppress tumor development and respond to cancer therapy (64). Furthermore, P53-mediated apoptosis is through transcriptional activation of proapoptotic target genes in the intrinsic mitochondrial pathway. The intrinsic P53-mediated apoptotic pathway involves the induction of the Bcl-2 family members such as Bax and the BH3-only proteins Bid, Puma, and Noxa. P53 induced Bax activation, which leads to cytochrome c release followed by caspase kinase activation and cellular apoptosis (65). Moreover, the expression of Bcl-2 family members, P53, and ROS generation associated with AKT has been revealed in previous studies (66, 67). As expected, the expression of XIAP was significantly down-regulated, while P53 significantly up-regulated in cells treated the Δ-Ru1/Dox combination, which is consistent with previous studies.

**Figure 7:**
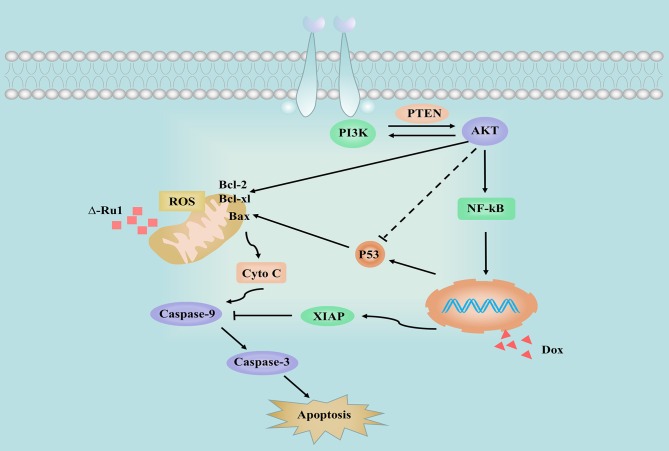
Schema showing the signaling pathways stimulated by different treatments in MCF-7 cells. The solid and dashed arrows illustrate the directed and indirect apoptotic signaling pathway upon treatment with Δ-Ru1, Dox, and the Δ-Ru1/Dox combination.

In summary, this study found that the Δ-Ru1/Dox combination has a synergistic effect against the cancer cell growth. The Δ-Ru1/Dox combination inhibits cell proliferation and induces apoptosis by increasing cellular ROS accumulation inactivating PI3K/AKT signaling pathway, including the expression of PTEN, NF-κB, XIAP, and P53 (Figure 7). These findings provide insights into the potential activity of the Δ-Ru1/Dox combination. It will encourage further investigations for alternative combination therapy using ruthenium complex combined with other chemotherapeutic agents, which shows a promising potential in clinical use.

## Data Availability Statement

All datasets generated for this study supporting the conclusions are included in the article and will be made available, without undue reservation, to any qualified researcher.

## Author Contributions

JW conceived the idea and designed the experiments. KL, YR, and DC performed the experiments and wrote the manuscript. ZZ and HB analyzed data and performed the statistical analysis. XH and AQ provided critical analysis and language editing. All the authors read and approved the final manuscript.

### Conflict of Interest

The authors declare that the research was conducted in the absence of any commercial or financial relationships that could be construed as a potential conflict of interest.
